# Optimal Control for a Superconducting Hybrid MagLev Transport System with Multirate Multisensors in a Smart Factory

**DOI:** 10.3390/s24020671

**Published:** 2024-01-21

**Authors:** Changhyun Kim

**Affiliations:** Department of Electronic Engineering, Kangnam University, Yongin-si 16979, Gyeonggi-do, Republic of Korea; chkim@kangnam.ac.kr

**Keywords:** magnetic levitation, superconducting hybrid EMS, multisensor fusion, in-track type EMS, smart factory

## Abstract

Recently, magnetic levitation systems have been applied and studied in various industrial fields. In particular, in-tracktype magnetic levitation conveyor systems are actively studied since they can effectively minimize electromagnetic effects in processes that require a highly clean environment. In this type of system, diverse and multiple sensors are structurally required so that the control performance of an integrated system is primarily governed by the slowest measuring sensor. This paper proposes a multisensor fusion compensator to integrate the outputs obtained from various sensors into one output with the single fastest time rate. Since the state of the system is estimated at a fast time rate, the optimal controller also guarantees fast performance and stability. The computation of electromagnetic fields and the control performance of the considered superconducting hybrid system were analyzed using a computer simulation based on finite element methods.

## 1. Introduction

Recently, magnetic levitation (MagLev) systems have been extensively researched for transporting logistics in various manufacturing plants as well as traditional transportation methods such as trains [1,2,3,4]. The studied MagLev systems are being considered to replace the existing belt-type conveyor system in the semiconductor and OLED display panel production process [3,4,5,6,7]. These processes require a highly clean environment because fine dust has a significant impact on production yield [8,9]. Therefore, the MagLev conveyor system, which does not generate contact dust, is more suitable than the traditional electric motor conveyor belt system [9,10].

This type of MagLev logistics transport system is divided into an on-board type and an in-track type depending on the location of the power device for electromagnetic suspension (EMS) [2,3,4]. In this paper, we consider an attracted superconducting hybrid (SH) electromagnetic suspension (EMS) [10,11]. This type is suitable for the above manufacturing process because all electromagnetic devices are installed on the track, so the electromagnetic impact of the system on transport logistics such as wafers and display panels is relatively low [12,13,14]. Additionally, the SH-EMS system allows for fast current control due to the lower electrical resistance of the superconductor compared to conventional formats, making it easy to compensate for the inherent instability of the MagLev system [15,16,17].

In the in-track magnetic levitation system considered in this paper, all sensors are installed on the track rather than on the vehicle, so multiple heterogeneous sensors are installed along the vehicle’s transport path [2,8,9]. Controlling system inputs requires processing and implementing multiple measured outputs from these sensors simultaneously. Various sensors are applied inside and outside the track and vehicle to measure output signals such as current, voltage, levitation gap, and propulsion position [2,8,9]. Typically, the control performance of the entire system is governed by the slowest measurement sensor among multiple heterogeneous sensors [18,19,20]. In the context of a typical output feedback control system based on measurements from multiple sensors, the controller operates in synchronization with the sensor with the lowest frequency. When fusing values from multirate sensors, they are integrated by assuming that the values from high-frequency sensors are constant for a given sampling time. Despite improvements in controller processing speed, such as digital signal processors (DSPs), graphics processing units (GPUs), and microprocessors, sensor processing often lags behind. If there are multiple sensors that need to process large amounts of data, such as image-processing sensors and LiDAR sensors, their processing speeds are different. In this case, the performance of the integrated control system is degraded due to the slow processing speed of the sensor. Therefore, the combination and integration of multiple sensors is one of the most important design elements to improve system control performance.

The in-track magnetic levitation logistics transport system discussed in this paper has various types of sensors and electrical components mounted on the track to increase status measurement accuracy, so numerous sensors operate simultaneously depending on the logistics transport distance. This paper proposes a model-based optimal state estimator and controller so that the integrated controller does not depend on the output of the slowest operating sensor. Unlike existing systems, the proposed system shows performance similar to a system with the fastest operating sensors because it can integrate and estimate the state even at interval times without measured output from the system model. The proposed controller allows all outputs to be integrated to achieve a fast sampling time, allowing good control results even for sensors of low quality. Through this, not only can the overall cost of the sensor system be reduced but total control performance can also be improved. The performance-stability of the proposed system is guaranteed by mathematical proof.

The proposed method estimates the state with the fastest sampling time based on a mathematical model of the system by combining multiple outputs with different data-processing times. The proposed compensator consists of two parts: a state estimator and a feedback controller [21]. The state estimator estimates the state according to the operation time of the feedback controller through a uniform time lifting operation based on the system model of the outputs measured with different sampling times [22,23]. By fusing several different heterogeneous sensors into one fast sensing time, the proposed state estimator can improve the control performance of sensors that operate slowly [24,25]. The state estimator and feedback control gain of the multisensor fusion systems are determined by solving the Riccati equation based on two optimal control techniques [26,27,28]. The designed estimator minimizes the mean square error for measurement and state errors using a white sequence noise process with known covariance and zero cross-correlation [21]. The feedback controller is optimally designed to minimize the quadratic figure of merit depending on the input and state [21]. As a result, the designed compensator ensures standard bounded performance of single time update system converted to uniform time lifting [18,19,20].

The proposed method is expected to be suitable for an in-track system where many sensors must be installed along the line extension considered in this paper. The effectiveness of the proposed method is verified through numerical simulations.

## 2. Fundamental Analysis of SH-MagLev Systems

**Figure 1:**
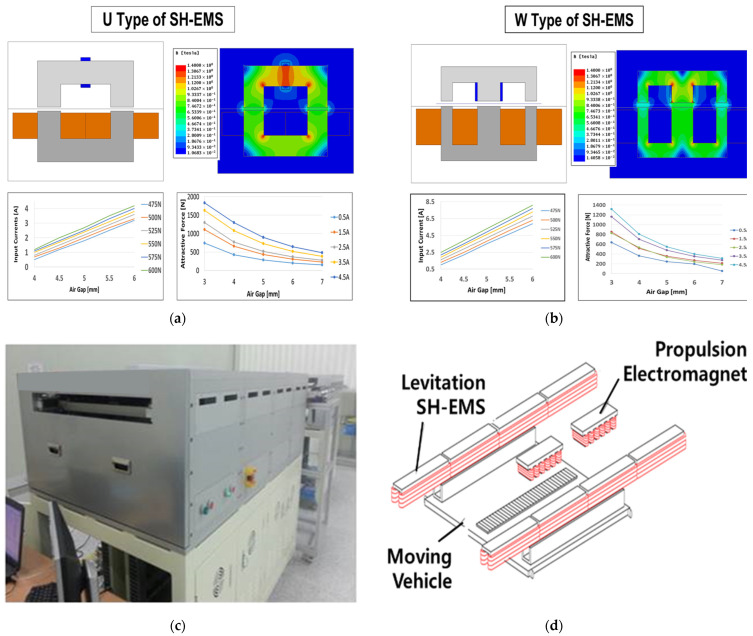
(**a**,**b**) show the results of an electromagnetic field analysis of levitational U-type and W-type SH-EMS systems, considering the air gaps, input currents and attractive forces of the SH-EMS systems using a finite element analysis. (**c**,**d**) show the module of the MagLev conveyor system and the structure of the moving vehicle and track [2,9].

Figure 1 shows the results of a finite element method (FEM) analysis of electromagnetic fields by type and the structure of the MagLev conveyor system with SH-EMS [2,9]. These two types are most often applied when manufacturing EMS shapes including superconducting coils. The FEM analysis results for levitational gap distance, input current, and attraction force in this shape are illustrated in Figure 1.

In this paper, in-track U-type EMS was considered. The actual manufacturing appearance and structure of the moving vehicle are described in Figure 1c,d. This is a re-expression of existing research results, and a more detailed explanation can be found in [2,9].

The mathematical model of SH-EMS is linearized as third-order differential equation at the equilibrium point, ($z_{0},\;{\dot{z}}_{0},\;i_{0})$ such that the gravity of the Earth is canceled. The voltage equation can be neglected since the current control operates sufficiently faster than the levitation controller. The reduced-order model of the linearized system is described as (1), in which the parameters are the same as the superconducting coil applied in the existing paper [2], shown in Table 1.

(1) $$\begin{array}{c} \dot{x}\left(t\right)=Ax\left(t\right)+B_{w}F_{d}\left(t\right)+B_{u}u\left(t\right)\\ y_{c}\left(t\right)=C_{y}x\left(t\right) \end{array}$$

where $x\left(t\right)=\left[\begin{array}{c} x_{1}\left(t\right)\\ x_{2}\left(t\right) \end{array}\right],A=\left[\begin{array}{cc} 0 & 1\\ \frac{1}{M}\frac{\mu_{0}A_{p}\left(N_{SC}^{2}I_{SC}^{2}+{N^{2}i}_{0}^{2}\right)}{2z_{0}^{3}} & 0 \end{array}\right],B_{w}=\left[\begin{array}{c} 0\\ \frac{1}{M} \end{array}\right],B_{u}=\left[\begin{array}{c} 0\\ -\frac{1}{M}\frac{\mu_{0}N^{2}A_{p}}{2}\frac{i_{0}}{z_{0}^{2}} \end{array}\right],C_{y}=\left[\begin{array}{cc} 1 & 0 \end{array}\right],x_{1}\left(t\right)=z\left(t\right)-z_{0}$, $x_{2}\left(t\right)=\dot{z}\left(t\right)-{\dot{z}}_{0}$, $u\left(t\right)=i\left(t\right)-i_{0}$.

## 3. Proposed Sensor Fusion Control Method

### 3.1. Sampled-Date System with Multirate Sensors

The state equation of the general sampled-data systems with sampling time is

(2) $$\begin{array}{c} x_{d}\left(k+1\right)=\Phi x_{d}\left(k\right)+\Gamma_{w}w_{d}\left(k\right)+\Gamma_{u}u_{d}\left(k\right)\\ z_{d}\left(k\right)=C_{z}x_{d}\left(k\right)+D_{zw}w_{d}\left(k\right)+D_{zu}u_{d}\left(k\right)\\ y_{d}\left(k\right)=C_{y}x_{d}\left(k\right)+D_{yw}w_{d}\left(k\right)+D_{yu}u_{d}\left(k\right) \end{array}$$

where $k=t/T_{0}$, $x_{d}(k)$, $w_{d}(k)$, $u_{d}(k)$, $z_{d}(k)$, and $y_{d}(k)$ are the discrete sample number, system state, exogenous inputs (disturbance $F_{d}(t)$, noise $n\left(t\right),$ and reference *r*), control input, cost signal to be controlled, and system outputs, respectively [9,21,29]. Equation (2) is a general system equation in the field of optimal control, and the parameters of SH-EMS model Equation (2) are explained as follows. The system matrices of the discretized SH-EMS system are $\Phi =e^{AT_{0}}$, $\Gamma_{w}=\int_{0}^{T_{d}}e^{A\left(T_{0}-t\right)}dtB_{w}$, $\Gamma_{u}=\int_{0}^{T_{d}}e^{A\left(T_{0}-t\right)}dtB_{u}$. Without a loss of generality, it can be assumed that noises are uncorrelated zero-mean white noise such that $D_{yu}$, ${\Gamma_{w}}^{T}D_{yw},$ and ${C_{z}}^{T}D_{zu}$ are zero matrices [18,21,29,30].

In Equation (2), $\Gamma_{w}$ and $D_{yw}$ are related to system disturbance and output noise and become important factors in the design of the discrete state observer. The control gain is determined by the optimal solution according to the cost signal, $z_{d}\left(k\right)$. The control gain is determined by the optimal solution according to the cost signal.

**Figure 2:**
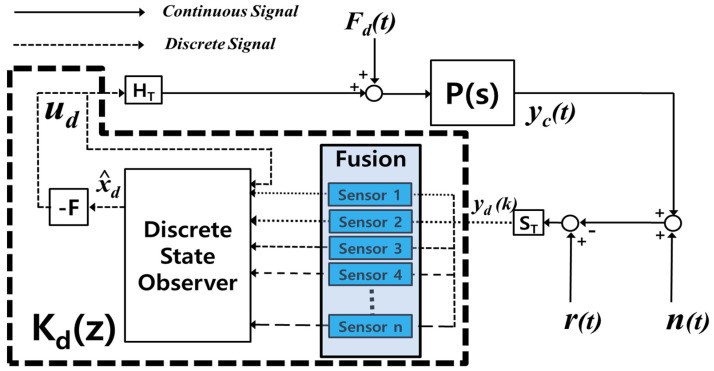
Structure of multirate sensor network system with discrete sensor fusion observer to estimate states with a fast-rate prediction time [29].

Figure 2 shows the structure of the multirate sensor fusion system for which the design method of $K_{d}\left(z\right)$ is proposed in this paper [29]. In Figure 2, solid and dotted lines represent continuous and discrete signals, respectively. The density of the dotted line indicates the measurement time of the sensors. P(s), $H_{T}$, and $S_{T}$ are system plants, holders, and samplers. The purpose of this paper is to design the optimal control gain, *F*, and a single discrete state observer that produce the estimated state, ${\hat{x}}_{d}(k)$, from sensors with different measurement times.

The system output is expressed as

(3) $$y_{d}\left(k\right)={\left[\begin{array}{ccccc} y_{1}\left(k\right) & y_{2}\left(k\right) & y_{3}\left(k\right) & \cdots & y_{n}\left(k\right) \end{array}\right]}^{T}.$$

For convenience, it is assumed that the measuring time,$T_{n}$, of the *n*th output, $y_{n}\left(k\right)$, has a relationship with a positive constant ratio,$R_{n}=T_{n}/T_{0}$, where $T_{0}$ represents the discrete-state sampling time of the system. Then the measured output is considered equal between measuring time intervals from sensors such that

(4) $$y_{n}\left(i*R_{n}+0\right)=y_{n}\left(i*R_{n}+1\right)=y_{n}\left(i*R_{n}+2\right)=\cdots \;=y_{n}\left(i*R_{n}+R_{n}-2\right)=y_{n}\left(i*R_{n}+R_{n}-1\right)\;i=0,1,\cdots$$

In the case of a system with *n* outputs, (4) of the outputs is expressed in detail as (5), where $R_{n}$ is a positive integer, *n*, as assumed above.

(5) $$\begin{array}{c} y_{d}\left(0\right)={\left[\begin{array}{cccccc} y_{1}\left(0\right) & y_{2}\left(0\right) & y_{3}\left(0\right) & \cdots & y_{n-1}\left(0\right) & y_{n}\left(0\right) \end{array}\right]}^{T},\\ y_{d}\left(1\right)={\left[\begin{array}{cccccc} y_{1}\left(1\right) & y_{2}\left(0\right) & y_{3}\left(0\right) & \cdots & y_{n-1}\left(0\right) & y_{n}\left(0\right) \end{array}\right]}^{T},\\ y_{d}\left(2\right)={\left[\begin{array}{cccccc} y_{1}\left(2\right) & y_{2}\left(2\right) & y_{3}\left(0\right) & \cdots & y_{n-1}\left(0\right) & y_{n}\left(0\right) \end{array}\right]}^{T},\\ y_{d}\left(3\right)={\left[\begin{array}{cccccc} y_{1}\left(3\right) & y_{2}\left(2\right) & y_{3}\left(3\right) & \cdots & y_{n-1}\left(0\right) & y_{n}\left(0\right) \end{array}\right]}^{T},\\ \vdots \\ y_{d}\left(k-2\right)={\left[\begin{array}{cccccc} y_{1}\left(k-2\right) & y_{2}\left(k-3\right) & y_{3}\left(k-2\right) & \cdots & y_{n-1}\left(0\right) & y_{n}\left(0\right) \end{array}\right]}^{T},\\ y_{d}\left(k-1\right)={\left[\begin{array}{cccccc} y_{1}\left(k-1\right) & y_{2}\left(k-1\right) & y_{3}\left(k-2\right) & \cdots & y_{n-1}\left(k-1\right) & y_{n}\left(0\right) \end{array}\right]}^{T},\\ y_{d}\left(k\right)={\left[\begin{array}{cccccc} y_{1}\left(k\right) & y_{2}\left(k-1\right) & y_{3}\left(k-2\right) & \cdots & y_{n-1}\left(k-1\right) & y_{n}\left(k\right) \end{array}\right]}^{T}. \end{array}$$

In the case of a system with *n* outputs, the outputs are expressed in detail as (5) where $R_{n}$ means a positive integer, *n*, as assumed above. The *n*th output, $y_{n}$, has the same value for *n* time steps between time intervals.

### 3.2. Multirate Sensor Fusion System

The following describes a discrete-time multirate system with multisampled outputs:

(6) $$\begin{array}{c} \begin{array}{c} x_{d}\left(k_{n},k_{n-1},\cdots ,k_{1},k_{0}+1\right) \end{array} \end{array}=\Phi \begin{array}{c} x_{d}\left(k_{n},k_{n-1},\cdots ,k_{1},k_{0}\right) \end{array}+\Gamma_{u}u_{d}\left(k_{n},k_{n-1},\cdots ,k_{1},k_{0}\right)$$

(7) $$y_{j}\left(k_{n},\cdots ,k_{j},0,\cdots ,0\right)=C_{y}x_{d}\left(k_{n},\cdots ,k_{j},0,\cdots ,0\right)$$

where $k_{n}=0,1,2,\ldots ,R_{n}$.

The discrete-state sampling time, *T*, is expressed by output sampling times as follows: $T=\sum_{i=0}^{n}k_{i}T_{i}$.

Applying the discrete-time lifting, we obtain

$$L_{\left(T_{0}\to T_{n}\right)}:l_{T_{n}}^{p}\to l_{T_{0}}^{p},\;\;\;1\leq p\leq \infty$$

leading to ${\tilde{u}}_{d}\left(k,0\right)=L_{\left(T_{0}\to T_{n}\right)}u_{d}\left(k_{n},k_{n-1},\cdots ,k_{1},k_{0}\right)$.

$${\tilde{u}}_{d}\left(k,0\right)=\left[\begin{array}{c} u_{d}\left(k_{n},0,0,\cdots ,0,0\right)\\ u_{d}\left(k_{n},0,0,\cdots ,0,1\right)\\ \vdots \\ u_{d}\left(k_{n},R_{n-1}-1,R_{n-2}-1,\cdots ,R_{1}-1,R_{0}-1\right) \end{array}\right],$$

where $k_{n}=\left(k_{n},k_{n-1},\cdots ,k_{0}\right)$, and 0 means that the previous multirate sampling time is zero [18,19]. The system state equation is derived as (8) by using a lifting operation.

(8) $$\begin{array}{c} x_{d}\left(k_{n},k_{n-1}+1\right)=\Phi \begin{array}{c} x_{d}\left(k_{n},k_{n-1}\right) \end{array}+\Gamma_{u}u_{d}\left(k_{n}\right)\\ =\Phi \begin{array}{c} \left(\Phi \begin{array}{c} x_{d}\left(k_{n},k_{n-1}-1\right) \end{array}+\Gamma_{u}u_{d}\left(k_{n},k_{n-1}-1\right)\right) \end{array}+\Gamma_{u}u_{d}\left(k_{n}\right)\\ =\Phi^{2}\begin{array}{c} \begin{array}{c} x_{d}\left(k_{n},k_{n-1}-1\right) \end{array}+\Phi \Gamma_{u}u_{d}\left(k_{n},k_{n-1}-1\right) \end{array}+\Gamma_{u}u_{d}\left(k_{n}\right)\\ =\Phi^{3}\begin{array}{c} \begin{array}{c} \begin{array}{c} \begin{array}{c} x_{d}\left(k_{n},k_{n-1}-2\right) \end{array} \end{array} \end{array} \end{array}++\Phi^{2}\Gamma_{u}u_{d}\left(k_{n},k_{n-1}-2\right)+\Phi \Gamma_{u}u_{d}\left(k_{n},k_{n-1}-1\right)+\Gamma_{u}u_{d}\left(k_{n}\right)\\ \vdots \\ =\Phi^{k_{0}+1}\begin{array}{c} \begin{array}{c} \begin{array}{c} \begin{array}{c} x_{d}\left(k_{n},k_{n-1}-k_{0}\right) \end{array} \end{array} \end{array} \end{array}+\Phi^{k_{0}}\Gamma_{u}u_{d}\left(k_{n},k_{n-1}-k_{0}\right)+\Phi^{k_{0}-1}\Gamma_{u}u_{d}\left(k_{n},k_{n-1}-k_{0}+1\right)+\cdots +\Gamma_{u}u_{d}\left(k_{n}\right) \end{array}$$

For $T=-T_{0}+\sum_{i=0}^{n-1}R_{i}T_{i}$, the multirate sensor networked system (2) is represented as (9).

(9) $$\begin{array}{l} x_{d}\left(k_{n}+1,0\right)=\tilde{\Phi }x_{d}\left(k_{n},0\right)+{\tilde{\Gamma }}_{w}{\tilde{w}}_{d}\left(k,0\right)+\tilde{\Gamma_{u}}{\tilde{u}}_{d}\left(k,0\right)\\ \tilde{z}\left(k_{n},0\right)={\tilde{C}}_{z}x_{d}\left(k_{n},0\right)+{\tilde{D}}_{zw}{\tilde{w}}_{d}\left(k,0\right)+{\tilde{D}}_{zu}{\tilde{u}}_{d}\left(k,0\right)\\ y_{d}\left(k_{n},0\right)=C_{y}x_{d}\left(k_{n},0\right)+{\tilde{D}}_{yw}{\tilde{w}}_{d}\left(k,0\right) \end{array}$$

where

$$R_{m}=\prod_{i=0}^{n-1}R_{i},\;{\tilde{\Gamma }}_{w}=\left[\begin{array}{cccc} \Phi^{R_{m}-1}\Gamma_{w} & \Phi^{R_{m}-2}\Gamma_{w} & \cdots & \Gamma_{w} \end{array}\right],$$

$$\tilde{\Phi }=\Phi^{R_{m}},\;\tilde{\Gamma_{u}}=\left[\begin{array}{cccc} \Phi^{R_{m}-1}\Gamma_{u} & \Phi^{R_{m}-2}\Gamma_{u} & \cdots & \Gamma_{u} \end{array}\right],$$

$${\tilde{C}}_{z}=\left[\begin{array}{c} C_{z}\\ C_{z}\Phi \\ \vdots \\ C_{z}\Phi^{R_{m}-1} \end{array}\right],{\tilde{D}}_{zw}=\left[\begin{array}{cccc} D_{zw} & 0 & \cdots & 0\\ C_{z} & D_{zw} & \cdots & 0\\ \vdots & \vdots & \ddots & \vdots \\ C_{z}\Phi^{R_{m}-2}\Gamma_{w} & C_{z}\Phi^{R_{m}-1}\Gamma_{w} & \cdots & D_{zw} \end{array}\right],$$

$${\tilde{D}}_{zu}=\left[\begin{array}{cccc} D_{zu} & 0 & \cdots & 0\\ C_{z} & D_{zu} & \cdots & 0\\ \vdots & \vdots & \ddots & \vdots \\ C_{z}\Phi^{R_{m}-2}\Gamma_{u} & C_{z}\Phi^{R_{m}-1}\Gamma_{u} & \cdots & D_{zu} \end{array}\right],$$

$${\tilde{D}}_{yw}=\left[\begin{array}{cccc} D_{\mathrm{yw}} & 0 & \cdots & 0 \end{array}\right],\;{\tilde{D}}_{yu}=\left[\begin{array}{cccc} D_{\mathrm{yu}} & 0 & \cdots & 0 \end{array}\right].$$

### 3.3. Design Method of Multirate Sensor Fusion Compensator

The proposed output feedback compensator combines the measured values of sensors with multirate sampling times and minimizes the *H*_2_ norm of the closed-loop system [8]. The proposed *H*_2_ compensator is designed for a lifted sensor networked system (9) with exogenous input, ${\tilde{w}}_{d},$ and output, $\tilde{z},$ and is applied to a multirate system (2). The observer and state feedback gain matrices are obtained from the unique positive-definite solutions of the dual discrete algebraic Riccati equations (DARE) (10) and (11) [2,19,29,30,31]

(10) $$Y=\tilde{\Phi }Y{\;\tilde{\Phi }}^{T}-\tilde{\Phi }YC_{y}^{T}(C_{y}YC_{y}^{T}+R_{k}{)}^{-1}C_{y}Y{\;\tilde{\Phi }}^{T}+Q_{k}$$

(11) $$X={\tilde{\Phi }}^{T}X\;\tilde{\Phi }-{\tilde{\Phi }}^{T}X\;\tilde{\Gamma_{u}}({\tilde{\Gamma }}_{u}^{T}X\;\tilde{\Gamma_{u}}+R_{u}{)}^{-1}{\tilde{\Gamma }}_{u}^{T}X\;\tilde{\Phi }+Q_{u}$$

where $R_{k}={\tilde{D}}_{yw}{\tilde{D}}_{yw}^{T},Q_{k}={\tilde{\Gamma }}_{w}{\tilde{\Gamma }}_{w}^{T},R_{u}={\tilde{D}}_{zu}^{T}{\tilde{D}}_{zu},\;and\;Q_{u}={\tilde{C}}_{z}^{T}{\tilde{C}}_{z}$.

From the solutions, the estimator gain, $\tilde{L},$ and state feedback gain, $\tilde{F}$, are obtained using (12) and (13).

(12) $$\tilde{L}=\tilde{\Phi }YC_{y}^{T}(C_{y}YC_{y}^{T}+R_{k}{)}^{-1}$$

(13) $$\tilde{F}=({\tilde{\Gamma }}_{u}^{T}X\;\tilde{\Gamma_{u}}+R_{u}{)}^{-1}{\tilde{\Gamma }}_{u}^{T}X\tilde{\Phi }$$

The proposed multirate sensor fusion compensator consists of a state estimator and a lifted state feedback control.

The state feedback control for the lifted state system is determined from (13) as ${\tilde{u}}_{d}\left(k,0\right)=-\tilde{F}{\hat{x}}_{d}\left(k,0\right)$ such that the control input, $u_{d}\left(k\right)$, is applied to the discrete system (2) according to the microcontroller’s fast update time to satisfy the following:

(14) $$u_{d}\left(k_{n},k_{n-1},\cdots ,k_{1},k_{0}\right)=-\tilde{F}{\hat{x}}_{d}\left(k_{n},k_{n-1},\cdots ,k_{1},k_{0}\right)$$

(15) $$\tilde{\Phi }-\tilde{\Gamma_{u}}\tilde{F}={\left(\Phi -\Gamma_{u}F\right)}^{R_{m}}$$

The stability of the proposed state feedback controller is guaranteed because Lyapunov’s stable condition (16) is satisfied by a Riccati equation, (11), similar to the general LQR (Linear Quadratic Regulator) compensator.

(16) $${\left(\tilde{\Phi }-\tilde{F}\tilde{\Gamma_{u}}\right)}^{T}X\;\left(\tilde{\Phi }-\tilde{F}\tilde{\Gamma_{u}}\right)-X<0$$

The fast-rate estimated state ${\hat{x}}_{d}\left(k\right)$ is obtained through the prediction and correction update process shown below.

Prediction update:

(17) $${\bar{x}}_{d}\left(k+1\right)=\Phi {\hat{x}}_{d}\left(k\right)+\Gamma_{u}u_{d}\left(k\right)$$

Correction update:

(18) $${\hat{x}}_{d}\left(k_{n},0\right)={\bar{x}}_{d}\left(k_{n},0\right)+L\left(y_{d}\left(k_{n},0\right)-C_{y}{\bar{x}}_{d}\left(k_{n},0\right)\right)$$

The proposed state estimator predicts the states according to the fast-rate sampling time and corrects the predicted states at the multirate sampling time of each output.

Appling the discrete-time lifting leads to a lifted prediction model (19) [19].

(19) $${\bar{x}}_{d}\left(k_{n}+1,0\right)=\left(\Phi^{R_{m}}-\sum_{i=1}^{R_{m}}\left(\Phi^{i}LC_{y}\right)\right){\bar{x}}_{d}\left(k_{n},0\right)+\sum_{i=1}^{R_{m}}\Phi^{i}Ly_{d}\left(k\right)+\tilde{\Gamma_{u}}{\tilde{u}}_{d}\left(k,0\right)$$

The stability and noise rejection performance of the proposed state estimator are guaranteed if the correction gain, *L*, of the multirate estimator satisfies (12), with $\tilde{L}$ obtained from a Riccati equation, (10), similar to a general Kalman filter.

(20) $$\tilde{\Phi }-\tilde{\Phi }\tilde{L}C_{y}=\Phi^{R_{m}}-\sum_{i=1}^{R_{m}}\left(\Phi^{i}LC_{y}\right)$$

such that the final filter gain *L* is determined as (21), satisfying (20).

(21) $$L={\left(\sum_{i=1}^{R_{m}}\Phi^{i}\right)}^{-1}\tilde{\Phi }\tilde{L}$$

Although there is a difference between the closed-loop system matrix of the proposed estimator and the observer with the general Kalman filter, its robust stability can be inherited by setting it to satisfy Equation (21). This provides the advantages of the proposed method to guarantee stability and overcome the limitations of the general Kalman filter, which operates slowly.

### 3.4. Summary of Design Procedure

The optimal design procedure of the multirate multisensor discrete-time system for the proposed MagLev system is summarized below.

Step 1. Find a linearized model $P\left(s\right)$ of nonlinear SH-EMS systems, given system parameters such as coil turns, superconducting coil turns, the current of the superconducting coil, and so on.

Step 2. Specify an appropriate cost signal $z_{d}\left(k\right)$ with weighting matrices, $R_{k},\;Q_{k},R_{u},\;and\;Q_{u}$ to consider performance and robustness which the designer wants to be small.

Step 3. Set a generalized multirate discrete-time plant $T\left(z\right)$ with an observer-based controller using a lifting operator.

Step 4. Determine the optimal multirate estimator using a Kalman filter by solving the optimization problem described as the discrete algebraic Riccati Equation (10). If the system states are not well estimated, then return to step 2.

Step 5. Determine the optimal state feedback gain, $\tilde{F,}$ to minimize the *H*_2_ performance indices for the single-rate sampled data system by solving the discrete algebraic Riccati Equation (11).

Step 6. Check the response of the closed-loop system in the time and frequency domains, and then return to the step 5 if it is unsatisfactory.

**Figure 3:**
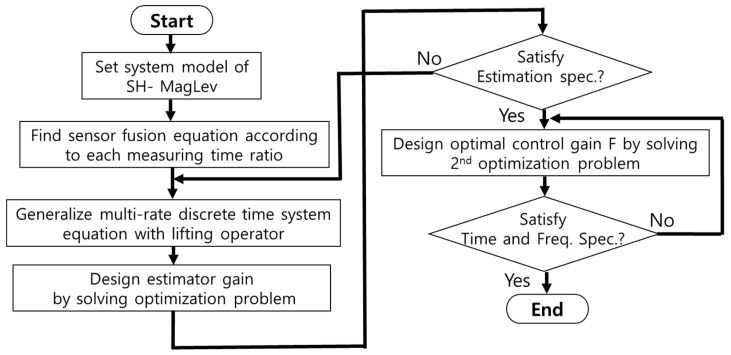
Design procedure of multirate sensor fusion compensator.

The proposed design procedure for a multirate sensor fusion compensator is summarized in Figure 3.

## 4. Simulation

For the simulation, an in-track MagLev conveyor system with the specifications shown in Table 1, using U-EMS in which the copper coil and the superconducting coil are installed in the track and the moving vehicle [2], was used.

The output of the system was set to be measured using sensors with multirate sampling times of 0.25 [ms], 0.5 [ms], and 2 [ms].

$$\begin{array}{c} y_{d}\left(0\right)={\left[\begin{array}{ccc} y_{1}\left(0\right) & y_{2}\left(0\right) & y_{3}\left(0\right) \end{array}\right]}^{T},\\ y_{d}\left(1\right)={\left[\begin{array}{ccc} y_{1}\left(1\right) & y_{2}\left(0\right) & y_{3}\left(0\right) \end{array}\right]}^{T},\\ y_{d}\left(2\right)={\left[\begin{array}{ccc} y_{1}\left(2\right) & y_{2}\left(2\right) & y_{3}\left(0\right) \end{array}\right]}^{T},\\ y_{d}\left(3\right)={\left[\begin{array}{ccc} y_{1}\left(3\right) & y_{2}\left(2\right) & y_{3}\left(0\right) \end{array}\right]}^{T},\\ y_{d}\left(4\right)={\left[\begin{array}{ccc} y_{1}\left(4\right) & y_{2}\left(4\right) & y_{3}\left(4\right) \end{array}\right]}^{T},\\ y_{d}\left(5\right)={\left[\begin{array}{ccc} y_{1}\left(5\right) & y_{2}\left(4\right) & y_{3}\left(4\right) \end{array}\right]}^{T},\\ \vdots \\ y_{d}\left(k-2\right)={\left[\begin{array}{ccc} y_{1}\left(k-2\right) & y_{2}\left(k-2\right) & y_{3}\left(k-4\right) \end{array}\right]}^{T},\\ y_{d}\left(k-1\right)={\left[\begin{array}{ccc} y_{1}\left(k-1\right) & y_{2}\left(k-2\right) & y_{3}\left(k-4\right) \end{array}\right]}^{T},\\ y_{d}\left(k\right)={\left[\begin{array}{ccc} y_{1}\left(k\right) & y_{2}\left(k\right) & y_{3}\left(k\right) \end{array}\right]}^{T}. \end{array}$$

White sequence noises with known error covariances of 100 and 0.0005 were applied to simulate the input disturbance, $F_{d}(t)$, and measurement error, n(t), of the system, respectively.

**Figure 4:**
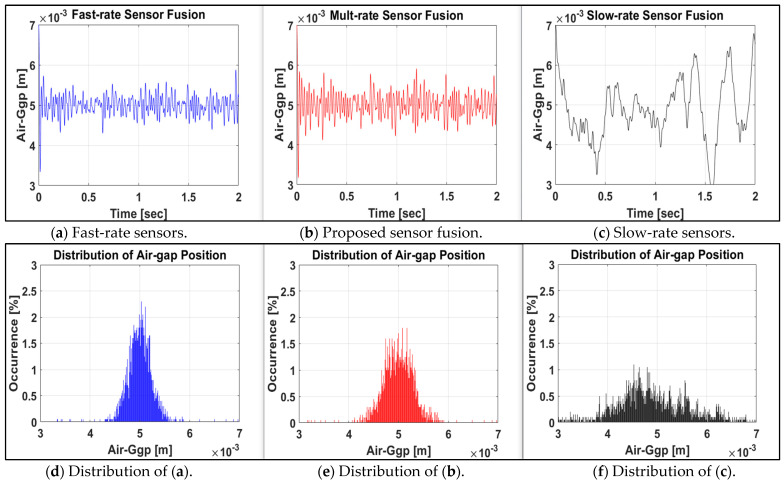
Time responses and percentage distributions of air-gap position of fast- (**a**,**d**) multi- (**b**,**e**) and slow-rate (**c**,**f**) systems.

As shown in Figure 4, the proposed multirate sensor fusion control system (b) provides similar results to the fast-rate sensor system (a), even though it includes the slowest measuring sensor system (c). The proposed method, the red line (e), shows a more concentrated distribution of the air gap position in the reference position, 5 mm, compared to the general control method (f), based on the slowest sensor’s operation. Even for sensors measuring the same output, the control response of the system with the slow measuring time fluctuates significantly in Figure 4c. In Figure 4b, the performance of systems with slow measurement times is improved by the proposed sensor fusion method. Figure 4d–f show how concentrated the responses are at the reference air gap position, 5 mm.

Figure 4d–f do not provide new information about the system’s time response, providing a visual insight into the advantages of the proposed method. The overall sharpness is more meaningful than the percentage of occurrence on the vertical axis in Figure 4d–f. Therefore, it can be confirmed that even the system containing some slow sensors can achieve control performance via the proposed method that is close to that of a fast-measuring system.

## 5. Conclusions

In this paper, we propose multisensor fusion and optimal control methods for superconducting hybrid MagLev conveyor systems in smart factories for semiconductor and display panel manufacturing. The proposed method can overcome the limitations of systems comprising sensors with slow measurement and processing times so that the total control performance is improved. Due to the structure of this system, in which the number of sensors is proportional to the distance of the logistics transfer track, this technique has the advantage of reducing the overall cost of the system and the performance impact of low-cost sensors.

The proposed multisensor fusion observer integrates its output with multiple measurement time rates into the fastest single time-rate output such that the predicted fast time states can be applied for optimal control. Because the proposed compensator is designed for a lifted sampled system, it guarantees inherited stability and norm-bound performance when applied to the original system. It was shown through a simulation that the proposed method exhibits similar performance to that of a fast-rate system despite the use of slow-measuring sensors.

It is expected that the proposed method will be well applied to in-track-type magnetic levitation systems that structurally require various sensors.

## Figures and Tables

**Table 1:** Parameters of SH-EMS.

Parameter	Description	Value
*N*	Coil Turns	730 turns
*N_SC_*	Superconducting Coil Turns	100 turns
*A_p_*	Pole Area of Coil	0.005 (m^2^)
*g*	The Gravity of Earth	9.80665 (m/s^2^)
*μ*_0_	Vacuum Magnetic Permeability	$4\pi \times 10^{-7}$ (H/m)
*M*	Mass of the Levitated Vehicle	50 (kg)
*i*(*t*)	Current of Coil	(Amp)
*I_SC_*	Current of Superconducting Coil	3.6 (Amp)
*z*(*t*)	Gap Position	(m)
*F_a_*(*t*)	Attractive Force	(N)
*F_d_*(*t*)	Disturbance Force	(N)
*z_ref_*	Desired Gap Reference	0.005 (m)

## Data Availability

Data are contained within the article.
